# *MBOAT7* rs641738 TT genotype is associated with poorer survival in MASLD-related hepatocellular carcinoma

**DOI:** 10.3389/fonc.2026.1931260

**Published:** 2026-08-12

**Authors:** Marta Guariglia, Gian Paolo Caviglia, Silvia Gaia, Chiara Rosso, Emanuela Rolle, Francesca Saba, Eleonora Dileo, Gemma Martina Silvestri, Angelo Armandi, Patrizia Carucci, Elisabetta Bugianesi

**Affiliations:** 1 Department of Medical Sciences, University of Torino, Turin, Italy; 2 Gastroenterology Unit, Città della Salute e della Scienza—Molinette Hospital, Turin, Italy

**Keywords:** HCC, metabolic dysfunction-associated steatotic liver disease, overall survival, prognosis, single nucelotide polymorphisms

## Abstract

**Introduction:**

Genetic variants involved in lipid and glucose metabolism have been implicated in liver disease progression and hepatocellular carcinoma (HCC) development in patients with metabolic dysfunction-associated steatotic liver disease (MASLD). However, their prognostic role in patients with established HCC remains unclear. We aimed to investigate the association between MASLD-related genetic variants and overall survival (OS) in patients with MASLD-related HCC.

**Methods:**

We retrospectively evaluated 258 patients with MASLD-related HCC (median age: 73, 49–79 years; male sex: 86.8%); most patients had a diagnosis of early tumor (BCLC 0/A: n = 162; 62.8%). *PNPLA3* rs738409, *TM6SF2* rs58542926, *MBOAT7* rs641738, *GCKR* rs780094, and *HSD17B13* rs72613567 variants were genotyped. An independent cohort of viral-related HCC (n = 384) was analysed for exploratory comparison. Overall survival (OS) was evaluated using Kaplan-Meier analysis and multivariable Cox regression.

**Results:**

Among the investigated gene variants, only the *MBOAT7* rs641738 TT genotype was associated with reduced OS in patients with MASLD-related HCC compared to CC/CT carriers (19.8 vs. 32.2 months; p = 0.006). At multivariable analysis, *MBOAT7* rs641738 TT genotype retained a nominal association with poorer OS after adjustment for age, sex, tumor burden, and metabolic cofactors (aHR = 1.61, 95% CI 1.05–2.46; *p* = 0.028). Conversely, OS did not differ according to *MBOAT7* rs641738 genotype in the exploratory viral-related HCC comparison cohort (*p* = 0.449).

**Conclusions:**

*MBOAT7* rs641738 genotype was associated with poorer OS in patients with MASLD-related HCC. This finding was not observed in an exploratory viral-related HCC comparison cohort, but between-cohort differences limit etiological interpretation. These results should be considered hypothesis-generating and require external validation.

## 1 Introduction

Hepatocellular carcinoma (HCC) is the sixth most prevalent cancer and the third leading cause of cancer-related mortality worldwide, accounting for approximately 90% of primary liver malignancies. Its incidence continues to rise, with a growing proportion of cases now attributable to non-viral etiologies, particularly metabolic dysfunction-associated steatotic liver disease (MASLD) (1, 2).

The current prognostic assessment of patients with HCC is primarily based on the Barcelona Clinic Liver Cancer (BCLC) staging system, which incorporates tumor burden, liver function, and patient performance status (3). However, this approach may fail to capture relevant biological or molecular determinants that may contribute to the heterogeneity of clinical outcomes observed among patients classified within the same BCLC stage. Genetic determinants of HCC susceptibility and genetic modifiers of survival after HCC diagnosis may represent distinct biological processes, since post-diagnosis outcomes are influenced not only by carcinogenic pathways but also by residual liver disease, host metabolism, inflammation, tumor biology, and treatment feasibility.

Recent research has highlighted the role of genetics in liver disease progression and HCC development (4, 5). Single-nucleotide polymorphisms in genes involved in lipid metabolism, such as *Patatin-like phospholipase domain-containing 3* (*PNPLA3*), *Transmembrane 6 superfamily member 2* (*TM6SF2*), *Membrane-bound O-acyltransferase domain-containing 7* (*MBOAT7*), and *Glucokinase regulator* (*GCKR*), have been associated with increased susceptibility to MASLD, fibrosis, and HCC (6), while a loss-of-function variant in *Hydroxysteroid 17-beta dehydrogenase 13* (*HSD17B13*) appears to confer protection against liver disease progression (7). However, the impact of these susceptibility variants on survival after HCC diagnosis remains largely unexplored, and it is unclear whether they contribute to tumor aggressiveness or modulate disease trajectory beyond established clinical staging systems.

In this study, we investigated the prognostic impact of selected genetic variants involved in liver disease pathogenesis in a cohort of patients with MASLD-related HCC.

## 2 Methods

### 2.1 Patients and study design

This is a retrospective, single-center study including patients with MASLD-related HCC, consecutively recruited at the Liver Clinic of the Città della Salute e della Scienza – Molinette Hospital in Turin, Italy, between 2012 and 2024. Inclusion criteria were age ≥18 years, a confirmed diagnosis of HCC, evidence of MASLD as underlying liver disease, and the availability of frozen whole blood for genetic analysis. Patients with HCC at BCLC D stage were excluded from the study. No otherwise eligible patient was excluded because of unavailable biological samples.

As an exploratory comparison analysis, an additional cohort of patients with viral-related HCC evaluated at the same Institution during the same study period was analysed to assess whether the prognostic impact of selected genetic variants differed across etiologies.

MASLD was defined according to contemporary guideline criteria as the presence of liver steatosis in patients with at least one cardiometabolic risk factor and no other predominant causes of chronic liver disease, including significant alcohol consumption (8). Given the retrospective design of the study and the historical use of the term non-alcoholic fatty liver disease (NAFLD) during early enrolment years, patients originally classified as NAFLD-related HCC were reclassified according to the updated MASLD definition through systematic review of archived medical records (9); all patients included in the final cohort fulfilled MASLD criteria after review, and no patient was excluded because of the terminology update.

Liver cirrhosis was diagnosed based on histological assessment or by non-invasive criteria, including vibration-controlled transient elastography (FibroScan, Echosens, Paris, France) and/or by indirect signs of portal hypertension, such as the presence of abdominal collateral veins, platelet count <150 × 10^9^/L, or esophageal varices (10). HCC was diagnosed according to international guidelines, based on histology or typical imaging features; tumor staging followed the BCLC system (3). First-line treatment was categorized as curative-intent, disease-control therapy, or no treatment. Curative-intent treatment included liver transplant (LT), surgical resection, stereotactic body radiation therapy (SBRT), and thermal ablation; disease-control therapy included transarterial chemoembolization (TACE), transarterial radioembolization (TARE), and systemic therapy.

The study was conducted in accordance with the Declaration of Helsinki and approved by the Ethics Committee of A.O.U. Città della Salute e della Scienza di Torino—A.O. Ordine Mauriziano—A.S.L. Città di Torino (protocol code 0008732; 26 January 2022). Written informed consent was obtained from all patients enrolled.

### 2.2 Genotyping

Genomic DNA was isolated from whole blood samples stored at -80 °C using an automated extraction system (EZ1 DSP DNA Blood Kit, QIAGEN, Hilden, Germany), according to the manufacturer’s instructions. After the isolation, DNA concentration and purity were measured in each sample using a NanoDrop ND-1000 spectrophotometer (Thermo Fisher Scientific, Wilmington, DE, USA). *PNPLA3* rs738409 (C>G), *TM6SF2* rs58542926 (C>T), *MBOAT7* rs641738 (C>T), *GCKR* rs780094 (C>T), and *HSD17B13* rs72613567 (TA insertion) genotyping was performed by real-time allelic discrimination assay (TaqMan SNP Genotyping Assay, Applied Biosystems, Foster City, CA) using TaqMan SNP Genotyping Master Mix (Applied Biosystems) on the CFX96 real-time PCR system (Bio-Rad Laboratories, Hercules, CA, USA), as previously reported (11). Appropriate positive and negative controls were added in each real-time PCR experiment. Genotyping quality control included assessment of call rate for each variant and Hardy-Weinberg equilibrium testing using an exact test.

### 2.3 Statistical analysis

Categorical variables were expressed as absolute numbers (n) and percentages (%), while continuous variables as medians and interquartile ranges (IQR). Comparison between categorical variables was performed using the chi-square (χ^2^) test or Fisher’s exact test, as appropriate. The Mann-Whitney U test was used to assess differences in continuous variables between groups.

Survival analysis was performed using Kaplan-Meier curves, and differences between groups were tested using log-rank test. Correction for multiple testing across the five candidate variants was performed for genotype-stratified Kaplan-Meier/log-rank analyses using Bonferroni correction and the Benjamini-Hochberg false discovery rate (FDR) procedure.

Multivariable Cox regression was used to evaluate the association between selected variables and overall survival (OS); results were expressed as adjusted hazard ratios (aHR) and 95% CI. OS was defined as the time from HCC diagnosis to death from any cause. Patients alive at last available follow-up were censored at the date of last contact, and patients undergoing liver transplantation were censored at transplantation. Deaths unrelated to liver disease were counted as events.

A p-value <0.05 was considered statistically significant. All statistical analyses were conducted using MedCalc software, version 20.1 (MedCalc, Ostend, Belgium).

## 3 Results

### 3.1 Baseline clinical characteristics and genotype distribution

A total of 258 patients with MASLD-related HCC were retrospectively enrolled. The demographic, clinical, and biochemical characteristics of the cohort are summarized in **Table 1**. Median age was 73 (49–79) years, and 86.8% were male. Liver cirrhosis was diagnosed in 207 (80.2%) patients. Most patients (n = 162; 62.8%) presented an early HCC stage (BCLC 0 or A), while 57 (22.1%) were classified as intermediate stage (BCLC B), and 39 (15.1%) as advanced stage (BCLC C). Median observed follow-up was 12.3 months (IQR 5.5–23.8). Nearly three-quarters of patients [n = 185 (71.7%)] received at least one treatment for HCC after diagnosis; 124 (48.1%) patients received curative-intent treatment, 61 (23.6%) received disease-control therapy, while 73 (28.3%) received no treatment. Overall, 77 (29.8%) patients were under semestral surveillance at the time of HCC diagnosis.

**Table 1:** Demographic, clinical, and biochemical characteristics of the MASLD-related HCC cohort.

Variables	Patients (n = 258)
Age (years), median (IQR) [258]	73 (49–79)
Sex, M/F [258]	224/34
BMI, kg/m^2^ median (IQR) [242]	29 (26–32)
T2DM, n (%) [228]	159 (69.7%)
Obesity, n (%) [242]*	113 (46.7%)
Hypertension, n (%) [227]	145 (63.9%)
Liver cirrhosis, n (%) [258]	207 (80.2%)
AST (U/L), median (IQR) [212]	39 (27–56)
ALT (U/L), median (IQR) [219]	31 (22–46)
γGT (U/L), median (IQR) [212]	93 (44–200)
Platelet count (x10^9^/L), median (IQR) [243]	143 (92–185)
INR median (IQR) [225]	1.16 (1.01–1.28)
Albumin (g/dL), median (IQR) [219]	3.9 (3.4–4.2)
Total bilirubin (mg/dL), median (IQR) [236]	1.0 (0.6–1.5)
AFP (ng/mL), median (IQR) [258]	5.2 (3.2–17.8)
BCLC staging [258]	
- 0, n (%) - A, n (%) - B, n (%) - C, n (%)	50 (19.4%) 112 (43.4%) 57 (22.1%) 39 (15.1%)
First-line treatment [258]**	
- Curative-intent, n (%) - Disease-control, n (%) - No treatment, n (%)	124 (48.1%) 61 (23.6%) 73 (28.3%)

Numbers in brackets indicate the number of patients with available data.

*Defined for BMI ≥ 30 kg/m^2^.

**Curative-intent treatment included LT (n = 10), surgical resection (n = 13), SBRT (n = 24), and thermal ablation (n = 77); disease-control therapy included TACE (n = 36), TARE (n = 10), and systemic therapy (n = 15).

AFP, alpha-fetoprotein; ALT, alanine aminotransferases; AST, aspartate aminotransferases; BCLC, Barcelona Clinic Liver Cancer; BMI, body mass index; F, female; γGT, gamma-glutamyl transferase; HCC, hepatocellular carcinoma; INR, international normalized ratio; IQR, interquartile range; LT, liver transplant; M, male; n, number; SBRT, stereotactic body radiation therapy; T2DM, type 2 diabetes mellitus; TACE, transarterial chemoembolization; TARE, transarterial radioembolization.

Genotyping call rate was 100% for all variants; Hardy-Weinberg equilibrium results are reported in **Supplementary Table 1**. For *PNPLA3* rs738409, 29.8% of patients were homozygous for the G risk allele, while 31.8% were homozygous for the T allele at *GCKR* rs780094. For *MBOAT7* rs641738, 47.7% of patients were heterozygous (CT) and 25.2% were homozygous for the T allele (TT). In contrast, *TM6SF2* rs58542926, and *HSD17B13* rs72613567 risk variants were less frequent, with 0.4% and 4.7% homozygous carriers, respectively (**Supplementary Figure 1**). Patients’ features according to pathogenic variants are reported in **Supplementary Tables 2–6**. Cumulative variants distribution is reported in **Supplementary Table 7**.

### 3.2 Association between genetic variants and overall survival in patients with MASLD-related HCC

**Figure 1:**
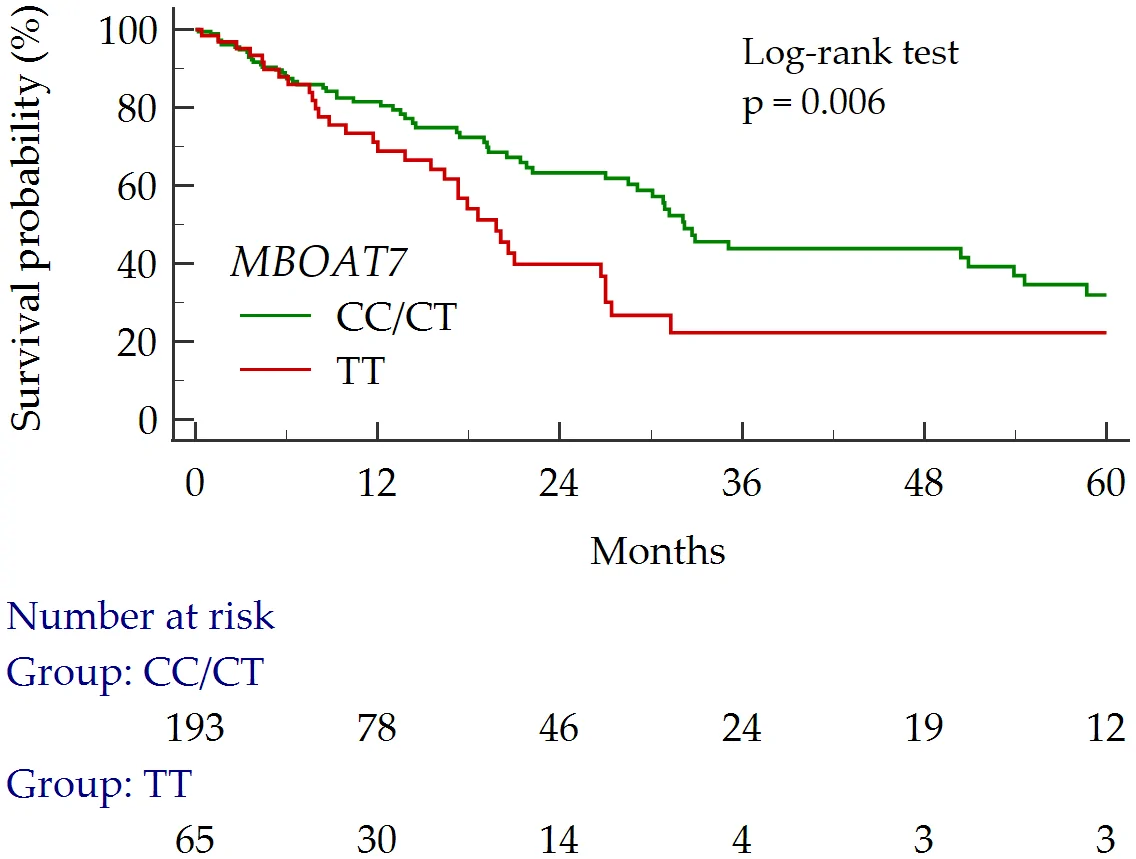
OS in patients with MASLD-related HCC according to MBOAT7 rs641738 genotype. Kaplan-Meier curves depict OS according to MBOAT7 rs641738 genotype using a recessive genetic model (CC/CT vs. TT). OS was defined as the time from HCC diagnosis to death from any cause; patients were censored at liver transplantation or at last FU. Differences between groups were assessed using the log-rank test.

The median OS was 28.5 (95% CI 21.4–32.2) months. As expected, more advanced BCLC stages were significantly associated with reduced OS (**Supplementary Figure 2**). Kaplan–Meier curves were generated for each genetic variant using genotype-based comparisons, with patients grouped according to established genetic models for each specific variant. Among the investigated polymorphisms, only *MBOAT7* rs641738 was significantly associated with OS. During follow-up, death occurred in 35 of 65 patients carrying the MBOAT7 rs641738 TT genotype (53.8%) and in 64 of 193 CC/CT carriers (33.2%). Consistently, patients homozygous for the minor allele (TT) had a median OS of 19.8 (95% CI 15.5–27.0) months, compared to 32.2 (95% CI 28.5–53.9) months in those heterozygous or homozygous for the major allele (*p* = 0.006) (**Figure 1**). This association remained significant after correction for testing the five candidate variants (Bonferroni-adjusted *p* = 0.030; FDR-adjusted *p* = 0.030). No significant differences in OS were observed for the other variants (**Supplementary Figure 3**).

In sensitivity analyses using alternative genotype codings, the association between *MBOAT7* rs641738 and OS was also observed when the three genotypes were analysed separately (CC vs. CT vs. TT; log-rank *p* = 0.019) and in an ordered genotype trend analysis (CC = 0, CT = 1, TT = 2; *p* = 0.010), whereas the dominant model (CT/TT vs. CC) was not significant (p = 0.119) (**Supplementary Table 8**). These findings suggest that the survival difference was mainly driven by TT homozygotes.

At multivariable Cox regression analysis, the *MBOAT7* rs641738 TT genotype retained a nominal association with poorer OS (aHR = 1.61, 95% CI 1.05–2.46; *p* = 0.028), after adjustment for age, sex, tumor burden (BCLC), and metabolic cofactors (T2DM and/or obesity) (**Table 2**). This association was broadly consistent across progressive sensitivity models exploring alternative adjustment strategies and model specifications (**Supplementary Table 9**; **Supplementary Figure 4**).

**Table 2:** Multivariable Cox regression model assessing the association between *MBOAT7* rs641738 genotype, demographic features, tumor burden, and metabolic factors with overall survival.

Variables	aHR, 95% CI	*p* value
*MBOAT7* rs641738 TT	1.61, 1.05–2.46	0.028
Age (years)	1.00, 0.98–1.03	0.659
Male sex	1.17, 0.58–2.35	0.664
BCLC stage	2.26, 1.79–2.85	<0.001
Obesity and/or T2DM	0.81, 0.51–1.27	0.349

The model included baseline variables available at HCC diagnosis together with *MBOAT7* genotype. *MBOAT7* rs641738 TT was compared with CC/CT carriers. BCLC stage was entered as an ordinal variable. Obesity and/or T2DM was coded as a binary variable.

BCLC, Barcelona Clinic Liver Cancer; CI, confidence interval; aHR, adjusted hazard ratio; T2DM, type 2 diabetes mellitus.

### 3.3 Exploratory analysis in viral-related HCC

To explore whether the prognostic association observed in the MASLD cohort was also detectable in a different etiological context, an exploratory analysis was conducted in a cohort of patients with viral-related HCC (n = 384). The demographic, clinical, and biochemical characteristics of the cohort are summarized in **Supplementary Table 10**. The median age was 65 (57–74) years, and 288 (75.0%) patients were male. HCV infection was the predominant etiology (n = 320; 83.3%), while HBV accounted for 64 (16.7%) of cases. Most patients (n = 232; 60.4%) presented an early HCC stage (BCLC 0 or A), while 90 (23.4%) were classified as intermediate stage (BCLC B), and 62 (16.1%) as advanced stage (BCLC C). Overall, 80 (20.8%) patients carried the *MBOAT7* rs641738 TT genotype.

**Figure 2:**
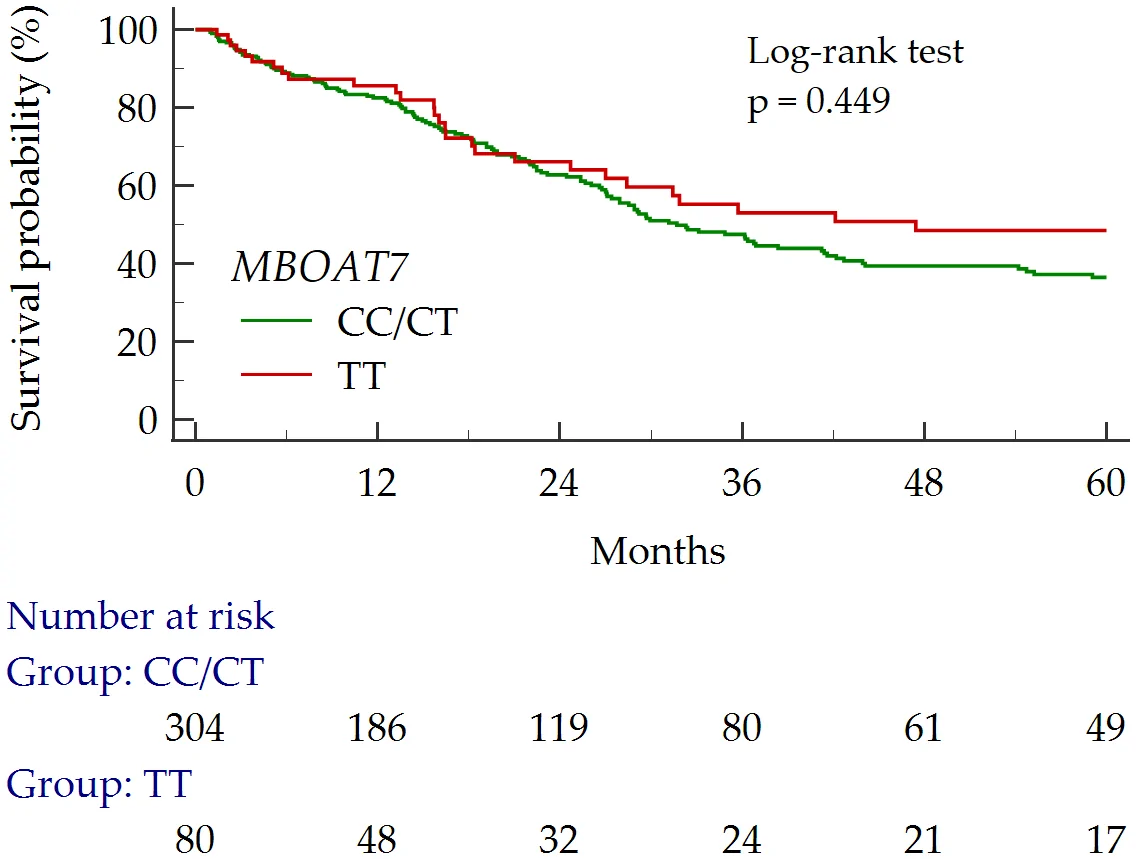
OS in patients with viral-related HCC according to MBOAT7 rs641738 genotype. Kaplan-Meier curves depict OS according to MBOAT7 rs641738 genotype using a recessive genetic model (CC/CT vs. TT). OS was defined as the time from HCC diagnosis to death from any cause; patients were censored at liver transplantation or at last FU. Differences between groups were assessed using the log-rank test.

Median observed follow-up was 16.5 months (IQR 7.0–38.0), while the median OS of the viral-related HCC cohort was 31.7 (95% CI 27.4–41.4) months. At Kaplan-Meier analysis, no significant differences in OS were observed between TT and CC/CT carriers (47.3, 95% CI 24.7–79.6 months vs 31.7, 95% CI 27.4–41.4 months; *p* = 0.449) (**Figure 2**). In a Cox regression model including an interaction term between liver disease etiology and *MBOAT7* rs641738 genotype, the interaction was statistically significant (aHR = 0.49, 95% CI 0.28–0.85; *p* = 0.012). However, this interaction was attenuated and was no longer statistically significant after adjustment for age, sex, BCLC stage, and first-line treatment, and in a further complete-case model additionally including liver function parameters (**Supplementary Table 11**).

## 4 Discussion

In our cohort of MASLD-related HCC patients, we observed that the *MBOAT7* rs641738 TT genotype was significantly associated with reduced OS. This association retained nominal significance after adjustment for demographic features, tumor burden, and metabolic cofactors, and was broadly consistent across progressive sensitivity models exploring alternative adjustment strategies. These findings raise the possibility that *MBOAT7* rs641738 may contribute to outcome heterogeneity in MASLD-related HCC beyond conventional clinical prognostic factors. Importantly, although the strongest signal was observed under a recessive model, the association was also supported by genotype-based sensitivity analyses, including a three-group comparison and an ordered genotype trend analysis. This suggests that the finding was not solely dependent on a *post hoc* dichotomization, although the survival difference appeared to be mainly driven by TT homozygotes. To the best of our knowledge, the prognostic impact of *MBOAT7* variants in patients with established HCC has not been previously investigated.

The lack of association between OS and the other established MASLD-related risk variants, including *PNPLA3*, *TM6SF2*, *GCKR*, and *HSD17B13*, may reflect the distinction between genetic determinants of MASLD/HCC susceptibility and genetic modifiers of outcome after HCC diagnosis (6, 7). These variants may primarily influence hepatic fat accumulation, chronic liver injury, fibrosis progression, and carcinogenesis, whereas survival after HCC onset is more strongly shaped by tumor stage, residual liver function, performance status, treatment feasibility, and tumor–host interactions (3). Therefore, the absence of a prognostic association for these variants in our cohort should not be interpreted as evidence against their pathogenic relevance, but rather as suggesting that their effect may be more related to liver disease progression and HCC development than to post-diagnosis survival.

*MBOAT7* rs641738 has received particular attention due to its functional implications in phospholipid remodelling and hepatic lipid metabolism. *MBOAT7* encodes a lysophosphatidylinositol acyltransferase that incorporates polyunsaturated fatty acids (preferentially arachidonic acid) into membrane phosphatidylinositols, which are specialized phospholipids that maintain cell membrane fluidity and support signal transduction pathways (12). *MBOAT7* loss-of-function impairs lipid remodelling, promoting the accumulation of saturated fatty acids, *de novo* lipogenesis, and lipotoxicity, which in turn promote hepatic inflammation, fibrogenesis, and carcinogenesis (13–15).

These biological effects may help explain the observed association between MBOAT7 variation and the poorer OS in MASLD-related HCC. Reduced MBOAT7 activity may increase the availability of free arachidonic acid, a precursor of eicosanoids such as prostaglandins and leukotrienes, and alter phosphatidylinositol composition, thereby affecting lipid-mediated inflammatory pathways and membrane-dependent signalling (16–18). Experimental evidence also suggests that reduced *MBOAT7* expression may enhance Toll-like receptor-mediated inflammatory responses and promote pro-inflammatory cytokine production, further supporting a potential link between altered lipid remodelling and inflammation-driven disease progression (19). However, because the present study did not include tissue-level or functional analyses, these mechanisms remain speculative and should not be interpreted as direct evidence of causality.

Finally, the exploratory analysis in patients with viral-related HCC further refines the interpretation of our main findings. Although the *MBOAT7* rs641738 variant has been associated with liver disease progression in chronic hepatitis B and C (20, 21), its association with poorer OS in our study was observed in the MASLD-related HCC cohort but not in the viral-related HCC comparison cohort. In MASLD, where lipotoxicity, insulin resistance, and chronic low-grade inflammation coexist (22, 23), altered lipid remodelling may represent a biologically plausible mechanism linking *MBOAT7* variation to disease trajectory. However, the absence of association in the unmatched viral-related HCC cohort may also reflect differences in baseline clinical characteristics, liver disease background, metabolic profile, tumor stage, and treatment allocation, rather than true etiological specificity.

Several limitations warrant consideration. First, the retrospective and single-center design may limit the generalizability of our findings. Second, external validation in independent cohorts of patients with MASLD-related HCC is lacking. In addition, although all patients were recruited at a single Italian tertiary referral center, residual population stratification cannot be completely excluded. Third, the study did not include tissue-level functional analyses, which prevents mechanistic inference regarding the biological pathways linking MBOAT7 variation to poorer OS. However, we must highlight that the study was conducted in a tertiary referral center using standardized diagnostic and therapeutic approaches and included a relatively large cohort of consecutively enrolled patients with homogeneous FU, which enhances the internal consistency of the analysis. Finally, although first-line treatment was evaluated in sensitivity analyses, subsequent therapies were not included as covariates because they represent post-diagnostic events and may introduce survivorship/immortal time bias. Moreover, given the long study period, potential changes in HCC treatment strategies over time could not be fully accounted for. Therefore, residual treatment-related confounding cannot be excluded, despite the broad consistency of the *MBOAT7* rs641738 association across progressive adjustment and sensitivity models.

In conclusion, *MBOAT7* rs641738 TT genotype was associated with reduced survival in patients with MASLD-related HCC and retained a nominal association with OS after adjustment for major baseline prognostic factors. This finding was not observed in the viral-related HCC comparison cohort, although between-cohort differences limit etiological interpretation. These results should be considered exploratory and require external validation.

## Data Availability

The datasets presented in this study can be found in online repositories. The names of the repository/repositories and accession number(s) can be found in the article/**Supplementary Material**.
