# Supplementary material for: MBOAT7 rs641738 TT genotype is associated with poorer survival in MASLD-related hepatocellular carcinoma
Source: Front Oncol. 2026 Aug 12;16:1931260. doi: 10.3389/fonc.2026.1931260 (PMC13506385; doi:10.3389/fonc.2026.1931260)
Supplement: Supplementary file 1 [file DataSheet1.docx]

**Supplementary Material**

Table of content…………………………………………………………………………………..page

Supplementary Table 1……………………………………………………………………………….2

Supplementary Table 2……………………………………………………………………………….3

Supplementary Table 3……………………………………………………………………………….4

Supplementary Table 4……………………………………………………………………………….5

Supplementary Table 5……………………………………………………………………………….6

Supplementary Table 6……………………………………………………………………………….7

Supplementary Table 7………………………………………...……………………….…………….8

Supplementary Table 8………………………………………...……………………….…………...10

Supplementary Table 9………………………………………...……………………….…………...11

Supplementary Table 10……………………………………...……………………….………….....12

Supplementary Table 11……………………………………...……………………….………….....13

Supplementary Figure 1…………………………………………………………………………….14

Supplementary Figure 2…………………………………………………………………………….15

Supplementary Figure 3…………………………………………………………………………….16

Supplementary Figure 4…………………………………………………………………………….17

**Supplementary Table 1.** Genotyping quality control and Hardy-Weinberg equilibrium analysis in the MASLD-related HCC cohort.

| **Variant** | **Genotype counts** | **Call rate** | **HWE exact *p* value** |
| --- | --- | --- | --- |
| *PNPLA3* rs738409 | CC 75 / CG 106 / GG 77 | 100% | 0.004 |
| TM6SF2 rs58542926 | CC 210 / CT 47 / TT 1 | 100% | 0.711 |
| *MBOAT7* rs641738 | CC 70 / CT 123 / TT 65 | 100% | 0.457 |
| GCKR rs780094 | CC 46 / CT 130 / TT 82 | 100% | 0.704 |
| *HSD17B13* rs72613567 | TT 167 / TAT 79 / TATA 12 | 100% | 0.557 |

Hardy-Weinberg equilibrium was assessed using an exact test. *PNPLA3* rs738409 deviated from Hardy-Weinberg equilibrium, possibly reflecting the case-only, disease-enriched design of the MASLD-related HCC cohort.

Abbreviations: HCC, hepatocellular carcinoma; HWE, Hardy-Weinberg equilibrium; MASLD, metabolic dysfunction-associated steatotic liver disease.

**Supplementary Table 2.** Demographic, clinical, and biochemical characteristics according to *PNPLA3* rs738409 genotype.

| **Variables** | **CC-CG**  **(n = 181)** | **GG**  **(n = 77)** | ***p* value*** |
| --- | --- | --- | --- |
| Age (years), median (IQR) [258] | 73 (64–79) | 74 (69–79) | 0.333 |
| Sex, M/F [258] | 164/17 | 60/17 | 0.006 |
| BMI, kg/m^2^ median (IQR) [242] | 29 (26–31) | 30 (25–33) | 0.489 |
| T2DM, n (%) [228] | 98 (61.6%) | 48 (69.6%) | 0.253 |
| Obesity, n (%) [242]** | 73 (44.0%) | 40 (52.6%) | 0.211 |
| Hypertension, n (%) [227] | 100 (62.9%) | 45 (66.2%) | 0.638 |
| Liver cirrhosis, n (%) [258] | 143 (79.0%) | 64 (83.1%) | 0.449 |
| AST (U/L), median (IQR) [212] | 38 (27–55) | 40 (27–59) | 0.515 |
| ALT (U/L), median (IQR) [219] | 30 (22–44) | 34 (23–52) | 0.318 |
| γGT (U/L), median (IQR) [212] | 91 (39–202) | 93 (55–190) | 0.824 |
| Platelet count (x10^9^/L), median (IQR) [243] | 146 (99–190) | 132 (75–182) | 0.093 |
| INR median (IQR) [225] | 1.16 (1.09–1.31) | 1.17 (1.09–1.25) | 0.445 |
| Albumin (g/dL), median (IQR) [219] | 3.9 (3.4–4.2) | 3.9 (3.5–4.4) | 0.124 |
| Total bilirubin (mg/dL), median (IQR) [236] | 1.0 (0.7–1.4) | 1.0 (0.7–1.7) | 0.538 |
| AFP (ng/mL), median (IQR) [258] | 5.2 (3.2–23.0) | 5.6 (3.5–12.8) | 0.847 |
| BCLC staging (n, %) [258]  - 0, n (%)  - A, n (%)  - B, n (%)  - C, n (%) | 33 (18.2%)  77 (42.5%)  41 (22.7%)  30 (16.6%) | 17 (22.1%)  35 (45.5%)  16 (20.8%)  9 (11.7%) | 0.234 |

Numbers in brackets indicate the number of patients with available data.

*calculated by Mann-Whitney test of the chi-squared test where appropriate.

**Defined for BMI ≥ 30 kg/m^2^.

Abbreviations: AFP, alpha-fetoprotein; ALT, alanine aminotransferases; AST, aspartate aminotransferases; BCLC, Barcelona Clinic Liver Cancer; BMI, body mass index; F, female; γGT, gamma-glutamyl transferase; HCC, hepatocellular carcinoma; INR, international normalized ratio; IQR, interquartile range; M, male; n, number; T2DM, type 2 diabetes mellitus.

**Supplementary Table 3.** Demographic, clinical, and biochemical characteristics according to *TM6SF2* rs58542926 genotype.

| **Variables** | **CC**  **(n = 210)** | **CT-TT**  **(n = 48)** | ***p* value*** |
| --- | --- | --- | --- |
| Age (years), median (IQR) [258] | 77 (69–82) | 77 (69–82) | 1.000 |
| Sex, M/F [258] | 186/24 | 38/10 | 0.083 |
| BMI, kg/m^2^ median (IQR) [242] | 29 (26–32) | 29 (27–32) | 0.917 |
| T2DM, n (%) [228] | 119 (64.0%) | 27 (64.3%) | 0.970 |
| Obesity, n (%) [242]** | 91 (46.0%) | 22 (50.0%) | 0.242 |
| Hypertension, n (%) [227] | 113 (61.1%) | 32 (76.2%) | 0.066 |
| Liver cirrhosis, n (%) [258] | 169 (80.5%) | 38 (79.2%) | 0.838 |
| AST (U/L), median (IQR) [212] | 40 (28–55) | 35 (23–59) | 0.346 |
| ALT (U/L), median (IQR) [219] | 32 (23–47) | 25 (21–39) | 0.077 |
| γGT (U/L), median (IQR) [212] | 98 (44–198) | 77 (40–202) | 0.546 |
| Platelet count (x10^9^/L), median (IQR) [243] | 140 (93–185) | 154 (83–190) | 0.835 |
| INR median (IQR) [225] | 1.15 (1.09–1.28) | 1.19 (1.13–1.28) | 0.278 |
| Albumin (g/dL), median (IQR) [219] | 3.9 (3.4–4.3) | 3.8 (3.3–4.1) | 0.401 |
| Total bilirubin (mg/dL), median (IQR) [236] | 1.0 (0.7–1.5) | 1.0 (0.7–1.5) | 0.502 |
| AFP (ng/mL), median (IQR) [258] | 5.1 (3.0–17.1) | 6.7 (3.9–19.3) | 0.185 |
| BCLC staging (n, %) [258]  - 0, n (%)  - A, n (%)  - B, n (%)  - C, n (%) | 39 (18.6%)  92 (43.8%)  44 (21.0%)  35 (16.7%) | 11 (22.9%)  20 (41.7%)  13 (27.1%)  4 (8.3%) | 0.330 |

Numbers in brackets indicate the number of patients with available data.

*calculated by Mann-Whitney test of the chi-squared test where appropriate.

**Defined for BMI ≥ 30 kg/m^2^.

Abbreviations: AFP, alpha-fetoprotein; ALT, alanine aminotransferases; AST, aspartate aminotransferases; BCLC, Barcelona Clinic Liver Cancer; BMI, body mass index; F, female; γGT, gamma-glutamyl transferase; HCC, hepatocellular carcinoma; INR, international normalized ratio; IQR, interquartile range; M, male; n, number; T2DM, type 2 diabetes mellitus.

**Supplementary Table 4**. Demographic, clinical, and biochemical characteristics according to *MBOAT7* rs641738 genotype.

| **Variables** | **CC-CT**  **(n = 193)** | **TT**  **(n = 65)** | ***p* value*** |
| --- | --- | --- | --- |
| Age (years), median (IQR) [258] | 73 (64–79) | 74 (67–79) | 0.545 |
| Sex, M/F [258] | 166/27 | 58/7 | 0.508 |
| BMI, kg/m^2^ median (IQR) [242] | 30 (27–32) | 28 (25–31) | 0.231 |
| T2DM, n (%) [228] | 106 (63.1%) | 40 (66.7%) | 0.622 |
| Obesity, n (%) [242]** | 92 (51.1%) | 21 (33.9%) | 0.019 |
| Hypertension, n (%) [227] | 97 (57.7%) | 48 (81.4%) | 0.001 |
| Liver cirrhosis, n (%) [258] | 155 (80.3%) | 52 (80.0%) | 0.957 |
| AST (U/L), median (IQR) [212] | 39 (27–56) | 40 (26–57) | 0.965 |
| ALT (U/L), median (IQR) [219] | 31 (21–46) | 31 (23–49) | 0.634 |
| γGT (U/L), median (IQR) [212] | 93 (39–194) | 92 (47–227) | 0.294 |
| Platelet count (x10^9^/L), median (IQR) [243] | 133 (90–189) | 152 (105–185) | 0.395 |
| INR median (IQR) [225] | 1.17 (1.08–1.29) | 1.15 (1.10–1.24) | 0.698 |
| Albumin (g/dL), median (IQR) [219] | 3.9 (3.4–4.3) | 3.9 (3.5–4.2) | 0.941 |
| Total bilirubin (mg/dL), median (IQR) [236] | 1.0 (0.7–1.5) | 1.0 (0.7–1.7) | 0.713 |
| AFP (ng/mL), median (IQR) [258] | 5.2 (3.0–16.7) | 6.1 (3.3–30.0) | 0.157 |
| BCLC staging (n, %) [258]  - 0, n (%)  - A, n (%)  - B, n (%)  - C, n (%) | 36 (18.7%)  90 (46.6%)  38 (19.7%)  29 (15.0%) | 14 (21.5%)  22 (33.8%)  19 (29.2%)  10 (15.4%) | 0.590 |

Numbers in brackets indicate the number of patients with available data.

*calculated by Mann-Whitney test of the chi-squared test where appropriate.

**Defined for BMI ≥ 30 kg/m^2^.

Abbreviations: AFP, alpha-fetoprotein; ALT, alanine aminotransferases; AST, aspartate aminotransferases; BCLC, Barcelona Clinic Liver Cancer; BMI, body mass index; F, female; γGT, gamma-glutamyl transferase; HCC, hepatocellular carcinoma; INR, international normalized ratio; IQR, interquartile range; M, male; n, number; T2DM, type 2 diabetes mellitus.

**Supplementary Table 5.** Demographic, clinical, and biochemical characteristics according to *GCKR* rs780094 genotype.

| **Variables** | **CC-CT**  **(n = 176)** | **TT**  **(n = 82)** | ***p* value*** |
| --- | --- | --- | --- |
| Age (years), median (IQR) [258] | 73 (65–79) | 75 (67–79) | 0.361 |
| Sex, M/F [258] | 154/22 | 70/12 | 0.638 |
| BMI, kg/m^2^ median (IQR) [242] | 29 (26–32) | 30 (27–32) | 0.241 |
| T2DM, n (%) [228] | 95 (60.5%) | 51 (71.8%) | 0.100 |
| Obesity, n (%) [242]** | 73 (44.0%) | 40 (52.6%) | 0.211 |
| Hypertension, n (%) [227] | 96 (61.5%) | 49 (69.0%) | 0.278 |
| Liver cirrhosis, n (%) [258] | 142 (80.7%) | 65 (79.3%) | 0.791 |
| AST (U/L), median (IQR) [212] | 35 (27–54) | 42 (31–59) | 0.081 |
| ALT (U/L), median (IQR) [219] | 30 (21–45) | 33 (24–50) | 0.245 |
| γGT (U/L), median (IQR) [212] | 93 (43–202) | 91 (46–188) | 0.890 |
| Platelet count (x10^9^/L), median (IQR) [243] | 139 (91–186) | 148 (96–185) | 0.867 |
| INR median (IQR) [225] | 1.16 (1.08–1.28) | 1.16 (1.10–1.28) | 0.683 |
| Albumin (g/dL), median (IQR) [219] | 3.9 (3.4–4.2) | 3.9 (3.4–4.3) | 0.904 |
| Total bilirubin (mg/dL), median (IQR) [236] | 0.9 (0.7–1.4) | 1.2 (0.7–1.7) | 0.032 |
| AFP (ng/mL), median (IQR) [258] | 5.2 (3.2–17.5) | 5.3 (3.2–21.1) | 0.519 |
| BCLC staging (n, %) [258]  - 0, n (%)  - A, n (%)  - B, n (%)  - C, n (%) | 33 (18.8%)  70 (39.8%)  45 (25.6%)  28 (15.9%) | 17 (20.7%)  42 (51.2%)  12 (14.6%)  11 (13.4%) | 0.161 |

Numbers in brackets indicate the number of patients with available data.

*calculated by Mann-Whitney test of the chi-squared test where appropriate.

**Defined for BMI ≥ 30 kg/m^2^.

Abbreviations: AFP, alpha-fetoprotein; ALT, alanine aminotransferases; AST, aspartate aminotransferases; BCLC, Barcelona Clinic Liver Cancer; BMI, body mass index; F, female; γGT, gamma-glutamyl transferase; HCC, hepatocellular carcinoma; INR, international normalized ratio; IQR, interquartile range; M, male; n, number; T2DM, type 2 diabetes mellitus.

**Supplementary Table 6.** Demographic, clinical, and biochemical characteristics according to *HSD17B13* rs72613567 genotype.

| **Variables** | **TT**  **(n = 167)** | **TAT-TATA**  **(n=91)** | ***p* value*** |
| --- | --- | --- | --- |
| Age (years), median (IQR) [258] | 73 (65–79) | 74 (64–79) | 0.733 |
| Sex, M/F [258] | 144/23 | 80/11 | 0.703 |
| BMI, kg/m^2^ median (IQR) [242] | 29 (26–31) | 29 (27–33) | 0.318 |
| T2DM, n (%) [228] | 90 (60.4%) | 56 (70.9%) | 0.117 |
| Obesity, n (%) [242]** | 71 (45.5%) | 42 (48.8%) | 0.621 |
| Hypertension, n (%) [227] | 101 (67.8%) | 44 (56.4%) | 0.091 |
| Liver cirrhosis, n (%) [258] | 140 (83.8%) | 67 (73.6%) | 0.050 |
| AST (U/L), median (IQR) [212] | 35 (27–54) | 45 (29–60) | 0.067 |
| ALT (U/L), median (IQR) [219] | 32 (21–45) | 31 (22–50) | 0.735 |
| γGT (U/L), median (IQR) [212] | 92 (39–193) | 93 (45–205) | 0.725 |
| Platelet count (x10^9^/L), median (IQR) [243] | 145 (100–185) | 132 (85–186) | 0.359 |
| INR median (IQR) [225] | 1.16 (1.08–1.26) | 1.17 (1.10–1.31) | 0.205 |
| Albumin (g/dL), median (IQR) [219] | 3.9 (3.4–4.3) | 3.9 (3.4–4.2) | 0.406 |
| Total bilirubin (mg/dL), median (IQR) [236] | 0.9 (0.7–1.4) | 1.2 (0.7–1.7) | 0.032 |
| AFP (ng/mL), median (IQR) [258] | 5.7 (3.2–22.8) | 5.1 (3.2–10.6) | 0.384 |
| BCLC staging (n, %) [258]  - 0, n (%)  - A, n (%)  - B, n (%)  - C, n (%) | 33 (19.8%)  67 (40.1%)  45 (26.9%)  22 (13.2%) | 17 (18.7%)  45 (49.5%)  12 (13.2%)  17 (18.7%) | 0.894 |

Numbers in brackets indicate the number of patients with available data.

*calculated by Mann-Whitney test of the chi-squared test where appropriate.

**Defined for BMI ≥ 30 kg/m^2^.

Abbreviations: AFP, alpha-fetoprotein; ALT, alanine aminotransferases; AST, aspartate aminotransferases; BCLC, Barcelona Clinic Liver Cancer; BMI, body mass index; F, female; γGT, gamma-glutamyl transferase; HCC, hepatocellular carcinoma; INR, international normalized ratio; IQR, interquartile range; M, male; n, number; T2DM, type 2 diabetes mellitus.

**Supplementary Table 7.** Distribution of combined *PNPLA3* rs738409, *TM6SF2* rs58542926, *MBOAT7* rs641738, *GCKR* rs780094, *and HSD17B13* rs72613567 genotypes in the study population.

| **Combination ID** | ***PNPLA3* (C>G)** | ***TM6SF2* (C>T)** | ***MBOAT7* (C>T)** | ***GCKR* (C>T)** | ***HSD17B13* (TA)** | **Patients**  **(n)** |
| --- | --- | --- | --- | --- | --- | --- |
| C1 | CC | CC | TT | CT | TT | 11 |
| C2 | CG | CC | CC | CT | TT | 10 |
| C3 | CC | CC | CT | CT | TT | 10 |
| C4 | CG | CC | CT | TT | TT | 9 |
| C5 | CG | CC | CT | CC | TT | 9 |
| C6 | CG | CC | CT | CT | TT | 8 |
| C7 | CC | CC | CT | CT | TAT | 8 |
| C8 | CC | CC | TT | TT | TT | 7 |
| C9 | GG | CC | CC | CT | TT | 7 |
| C10 | CG | CT | CT | CT | TT | 7 |
| C11 | CC | CC | CT | TT | TT | 7 |
| C12 | GG | CC | CT | TT | TT | 7 |
| C13 | CC | CC | CC | CC | TT | 6 |
| C14 | CG | CC | TT | CT | TT | 6 |
| C15 | GG | CC | CT | TT | TAT | 6 |
| C16 | CG | CC | CC | CC | TT | 6 |
| C17 | CG | CT | TT | CT | TT | 5 |
| C18 | CG | CC | CT | TT | TAT | 5 |
| C19 | GG | CC | CT | CT | TT | 5 |
| C20 | GG | CC | TT | CT | TT | 4 |
| C21 | CG | CC | CT | CT | TAT | 4 |
| C22 | GG | CC | CC | CT | TAT | 4 |
| C23 | CC | CC | CC | CT | TT | 4 |
| C24 | CG | CC | TT | CT | TAT | 4 |
| C25 | CC | CC | CC | TT | TT | 4 |
| C26 | GG | CC | CT | CT | TAT | 4 |
| C27 | CG | CC | CC | TT | TT | 4 |
| C28 | GG | CC | CT | CT | TATA | 3 |
| C29 | CG | CC | CC | CT | TAT | 3 |
| C30 | GG | CC | TT | TT | TAT | 3 |
| C31 | GG | CC | CC | TT | TT | 3 |
| C32 | CC | CC | CC | TT | TAT | 3 |
| C33 | GG | CC | CT | CC | TT | 3 |
| C34 | CG | CC | CT | TT | TATA | 3 |
| C35 | GG | CT | CT | CT | TAT | 3 |
| C36 | GG | CT | CT | CT | TT | 2 |
| C37 | GG | CT | CC | TT | TAT | 2 |
| C38 | GG | CT | TT | CT | TAT | 2 |
| C39 | GG | CC | CC | TT | TAT | 2 |
| C40 | GG | CT | CC | CT | TT | 2 |
| C41 | GG | CC | TT | CT | TAT | 2 |
| C42 | GG | CC | TT | CC | TT | 2 |
| C43 | CG | CT | TT | CT | TAT | 2 |
| C44 | GG | CT | TT | CT | TT | 2 |
| C45 | CC | CT | CT | CC | TAT | 2 |
| C46 | CG | CC | CC | TT | TAT | 2 |
| C47 | CG | CC | CT | CC | TAT | 2 |
| C48 | CG | CT | CT | CC | TAT | 2 |
| C49 | CC | CC | TT | TT | TAT | 2 |
| C50 | CG | CC | TT | CC | TT | 2 |
| C51 | CC | CC | CT | TT | TAT | 2 |
| C52 | GG | CC | TT | TT | TT | 1 |
| C53 | GG | CC | TT | CT | TATA | 1 |
| C54 | CC | CC | TT | CC | TT | 1 |
| C55 | GG | CC | TT | TT | TATA | 1 |
| C56 | CG | CT | CT | CC | TT | 1 |
| C57 | GG | CT | CC | CC | TT | 1 |
| C58 | CC | CT | CT | CT | TT | 1 |
| C59 | CC | CC | TT | CC | TATA | 1 |
| C60 | GG | CT | CT | CC | TT | 1 |
| C61 | CC | CC | CT | CT | TATA | 1 |
| C62 | CC | CC | CT | CC | TT | 1 |
| C63 | GG | CT | CT | TT | TT | 1 |
| C64 | CC | CC | CT | CC | TAT | 1 |
| C65 | GG | CT | CC | CT | TAT | 1 |
| C66 | CC | CT | TT | TT | TAT | 1 |
| C67 | CC | CT | CT | TT | TAT | 1 |
| C68 | CG | CT | CT | TT | TAT | 1 |
| C69 | CG | CC | CT | CT | TATA | 1 |
| C70 | GG | CC | CT | CC | TAT | 1 |
| C71 | CG | CC | TT | TT | TAT | 1 |
| C72 | CG | CC | TT | TT | TT | 1 |
| C73 | CG | CT | CC | CC | TAT | 1 |
| C74 | GG | CC | CC | CT | TATA | 1 |
| C75 | CG | CT | CC | CC | TT | 1 |
| C76 | CG | TT | CC | CT | TT | 1 |
| C77 | CG | CT | TT | TT | TT | 1 |
| C78 | CC | CC | CC | CT | TAT | 1 |
| C79 | CG | CT | TT | CC | TT | 1 |
| C80 | CG | CT | TT | CC | TAT | 1 |
| C81 | CG | CT | CT | TT | TT | 1 |
| C82 | CG | CT | CC | TT | TT | 1 |

Genotype combinations were derived from individual-level data. All observed combinations are reported, including those occurring in a single patient.

**Supplementary Table 8.** Sensitivity analyses evaluating the association between *MBOAT7* rs641738 genotype and overall survival according to alternative genotype codings.

| **Genetic model** | **Comparison** | **Test** | ***p* value** | **Interpretation** |
| --- | --- | --- | --- | --- |
| Recessive | TT vs CC/CT | log-rank | 0.006 | Main analysis |
| Three-genotype | CC vs CT vs TT | log-rank | 0.019 | Significant |
| Ordered genotype trend | CC = 0, CT = 1, TT = 2 | trend test | 0.010 | Significant trend |
| Dominant | CT/TT vs CC | log-rank | 0.119 | Not significant |
| Pairwise | CC vs CT | log-rank | 0.562 | Not significant |
| Pairwise | CC vs TT | log-rank | 0.014 | Significant |
| Pairwise | CT vs TT | log-rank | 0.036 | Significant |

The recessive model was used as the primary model. Additional genotype-based analyses were performed to assess whether the association was dependent on dichotomization. The dominant model was not significant, whereas the three-genotype comparison and ordered genotype trend analysis supported a TT-driven survival difference. In the recessive comparison, death occurred in 35/65 TT homozygotes (53.8%) and in 64/193 CC/CT carriers (33.2%). In the three-genotype analysis, death occurred in 22/70 CC patients (31.4%), 42/123 CT patients (34.1%), and 35/65 TT patients (53.8%).

**Supplementary Table 9.** Progressive sensitivity analysis assessing the association between *MBOAT7* rs641738 genotype and overall survival.

| **Model** | **Adjustment variables** | **n/events** | **aHR, 95% CI for MBOAT7 rs641738 TT** | ***p* value** |
| --- | --- | --- | --- | --- |
| Model 1 | Age, sex | 258/99 | 1.76, 1.16–2.67 | 0.008 |
| Model 2 | Age, sex, obesity and/or T2DM | 258/99 | 1.81, 1.19–2.76 | 0.006 |
| Model 3, primary baseline model | Age, sex, obesity and/or T2DM, BCLC stage | 258/99 | 1.61, 1.05–2.46 | 0.028 |
| Model 4, first-line treatment sensitivity model | Model 3 + first-line treatment | 258/99 | 1.57, 1.02–2.41 | 0.040 |
| Model 5, liver function complete-case sensitivity model | Model 4 + albumin, bilirubin, INR, platelet count | 202/76 | 1.79, 1.07–3.00 | 0.027 |
| Model 6, BCLC categorical sensitivity model | Age, sex, obesity and/or T2DM, BCLC as categorical variable | 258/99 | 1.57, 1.03–2.41 | 0.037 |
| Model 7, BCLC categorical + treatment sensitivity model | Model 6 + first-line treatment | 258/99 | 1.54, 1.00–2.36 | 0.050 |
| Model 8, AFP sensitivity model | Model 3 + log10(AFP) | 258/99 | 1.58, 1.03–2.41 | 0.036 |
| Model 9, AFP + treatment sensitivity model | Model 4 + log10(AFP) | 258/99 | 1.55, 1.01–2.37 | 0.047 |
| Model 10, AFP + liver function sensitivity model | Model 5 + log10(AFP) | 202/76 | 1.79, 1.07–2.99 | 0.027 |

*MBOAT7* rs641738 TT was compared with CC/CT carriers. In Models 1–5 and 8–10, BCLC stage was entered as an ordinal variable; in Models 6–7, BCLC stage was entered as a categorical variable. First-line treatment refers to the initial therapeutic strategy after HCC diagnosis and was categorized as curative-intent treatment, disease-control therapy, or no treatment, with curative-intent treatment as the reference category. AFP was log10-transformed because of its skewed distribution. Subsequent treatments received during follow-up were not included because they may introduce survivorship/immortal time bias.

Abbreviations: AFP, alpha-fetoprotein; aHR, adjusted hazard ratio; BCLC, Barcelona Clinic Liver Cancer; CI, confidence interval; HCC, hepatocellular carcinoma; INR, international normalized ratio; T2DM, type 2 diabetes mellitus.

**Supplementary Table 10.** Demographic, clinical, and biochemical characteristics of the viral-related HCC cohort.

| **Variables** | **Patients**  **(n = 384)** |
| --- | --- |
| Age (years), median (IQR) [384] | 65 (57–74) |
| Sex, M/F [384] | 288/96 |
| HCV, n (%) [384]  - active infection, n (%)  - eradicated, n (%) | 320 (83.3%)  184 (57.5%)  136 (42.5%) |
| HBV, n (%) [384]  - active infection, n (%)  - suppressed with NUCs, n (%) | 64 (16.7%)  18 (28.1%)  46 (71.9%) |
| Liver cirrhosis, n (%) [384] | 363 (94.5%) |
| AST (U/L), median (IQR) [358] | 52 (29–84) |
| ALT (U/L), median (IQR) [363] | 39 (23–73) |
| γGT (U/L), median (IQR) [344] | 66 (33–135) |
| Platelet count (x10^9^/L), median (IQR) [371] | 108 (76–185) |
| INR median (IQR) [365] | 1.16 (1.07–1.28) |
| Albumin (g/dL), median (IQR) [357] | 3.8 (3.4–4.3) |
| Total bilirubin (mg/dL), median (IQR) [364] | 1.0 (0.7–1.5) |
| AFP (ng/mL), median (IQR) [384] | 13.6 (4.5–63.0) |
| BCLC staging [384]  - 0, n (%)  - A, n (%)  - B, n (%)  - C, n (%) | 57 (14.8%)  175 (45.6%)  90 (23.4%)  62 (16.1%) |
| First-line treatment [384]*  - Curative-intent, n (%)  - Disease-control, n (%)  - No treatment, n (%) | 197 (51.3%)  103 (26.8%)  84 (21.9%) |

Numbers in brackets indicate the number of patients with available data.

*Curative-intent treatment included LT (n = 15), surgical resection (n = 17), SBRT (n = 19), thermal ablation (n = 144), and PEI (n = 2); disease-control therapy included TACE (n = 63), TARE (n = 12), and systemic therapy (n = 28).

Abbreviations: AFP, alpha-fetoprotein; ALT, alanine aminotransferases; AST, aspartate aminotransferases; BCLC, Barcelona Clinic Liver Cancer; F, female; γGT, gamma-glutamyl transferase; HBV, hepatitis B virus; HCC, hepatocellular carcinoma; HCV, hepatitis C virus; INR, international normalized ratio; IQR, interquartile range; LT, liver transplant; M, male; n, number; NUCs, nucleos(t)ide analogues; PEI, percutaneous ethanol injection; SBRT, stereotactic body radiation therapy; TACE, transarterial chemoembolization; TARE, transarterial radioembolization.

**Supplementary Table 11.** Cox regression models assessing the interaction between *MBOAT7* rs641738 genotype and liver disease etiology.

| **Variable** | **Model 1**  **aHR, 95% CI** | ***p* value** | **Model 2**  **aHR, 95% CI** | ***p* value** | **Model 3**  **aHR, 95% CI** | ***p* value** |
| --- | --- | --- | --- | --- | --- | --- |
| ***MBOAT7* rs641738 TT** | 1.79, 1.18–2.70 | 0.006 | 1.38, 0.91–2.09 | 0.132 | 1.28, 0.80–2.06 | 0.308 |
| **Viral etiology** | 1.02, 0.76–1.37 | 0.904 | 2.89, 1.97–4.23 | <0.001 | 3.21, 2.07–4.97 | <0.001 |
| **MBOAT7 rs641738 TT × viral etiology** | 0.49, 0.28–0.85 | 0.012 | 0.64, 0.37–1.13 | 0.125 | 0.71, 0.38–1.32 | 0.284 |
| Age, years | – | – | 1.02, 1.00–1.03 | 0.008 | 1.02, 1.00–1.03 | 0.009 |
| Male sex | – | – | 1.35, 0.99–1.83 | 0.058 | 1.48, 1.06–2.07 | 0.023 |
| BCLC stage | – | – | 2.23, 1.85–2.69 | <0.001 | 2.24, 1.81–2.76 | <0.001 |
| Disease-control therapy* | – | – | 2.05, 1.52–2.75 | <0.001 | 2.05, 1.49–2.82 | <0.001 |
| No treatment* | – | – | 3.05, 2.23–4.18 | <0.001 | 2.70, 1.91–3.81 | <0.001 |
| Albumin, g/dL | – | – | – | – | 0.70, 0.56–0.89 | 0.003 |
| Total bilirubin, mg/dL | – | – | – | – | 1.07, 0.99–1.17 | 0.099 |

The base interaction model (Model 1) included MBOAT7 rs641738 genotype, liver disease etiology, and their interaction term. The clinically adjusted model (Model 2) additionally included age, sex, BCLC stage, and first-line treatment. The liver function-adjusted model (Model 3) further included albumin and total bilirubin and was performed as a complete-case sensitivity analysis. MBOAT7 rs641738 TT was compared with CC/CT carriers. Etiology was coded as viral-related HCC versus MASLD-related HCC. BCLC stage was entered as an ordinal variable. First-line treatment was categorized as curative-intent treatment, disease-control therapy, or no treatment.

*Reference category: curative-intent treatment.

Abbreviations: aHR, adjusted hazard ratio; BCLC, Barcelona Clinic Liver Cancer; CI, confidence interval; HCC, hepatocellular carcinoma.

**Supplementary Figure 1.** Genotypic frequencies of *PNPLA3* rs738409, *TM6SF2* rs58542926, *MBOAT7* rs641738, *GCKR* rs780094, and *HSD17B13* rs72613567.

Bars indicate the number of patients carrying each genotype, with percentages calculated on the total MASLD-related HCC cohort (n = 258).

**Supplementary Figure 2.** OS in patients with MASLD-related HCC according to BCLC stage.

**Supplementary Figure 3.** OS in patients with MASLD-related HCC according to *PNPLA3* rs738409 (A), *TM6SF2* rs58542926 (B), *GCKR* rs780094 (C), and *HSD17B13* rs72613567 (D).

For *PNPLA3* rs738409, the median OS was 27.0 (95% CI 21.0–32.2) months in GG homozygotes versus 30.8 (95% CI 19.8–92.6) months in non-homozygotes (*p* = 0.249) (A). Regarding *TM6SF2* rs58542926, OS was 29.1 (95% CI 21.8–32.7) months in CC homozygotes and 27.4 (95% CI 17.4–50.4) months in those carrying at least one T allele (*p* = 0.681) (B). For *GCKR* rs780094, the OS was 26.7 (95% CI 19.3–32.9) months in TT homozygotes and 30.8 (95% CI: 27.0–35.1) months in others (*p* = 0.981) (C). Lastly, for *HSD17B13* rs72613567, OS was 27.4 (95% CI 20.6–35.1) months in major allele homozygotes and 30.8 (95% CI 17.3–58.7) months in carriers of the TA insertion (*p* = 0.832) (D).

**Supplementary Figure 4.** Forest plot showing the association between *MBOAT7* rs641738 TT genotype and overall survival across the primary Cox model and progressive sensitivity models.

Hazard ratios are shown for TT homozygotes compared with CC/CT carriers. Models correspond to those reported in Supplementary Table 9. Horizontal bars indicate 95% confidence intervals. AFP was log10-transformed because of its skewed distribution.
